# The High Prevalence of Vitamin D Deficiency and Its Related Maternal Factors in Pregnant Women in Beijing

**DOI:** 10.1371/journal.pone.0085081

**Published:** 2013-12-26

**Authors:** Shu Jun Song, Ling Zhou, Shaoyan Si, Junli Liu, Jinlian Zhou, Kai Feng, Jie Wu, Wenying Zhang

**Affiliations:** 1 Department of Pathology and Experimental Medicine, 306 Hospital of PLA, Beijing, P.R. China; 2 Department of Obstetrics and Gynecology, 306 Hospital of PLA, Beijing, P.R. China; 3 Department of Outpatient, 306 Hospital of PLA, Beijing, P.R. China,; University of Southampton, United Kingdom

## Abstract

Maternal vitamin D deficiency has been suggested to influence fetal and neonatal health. Little is known about vitamin D status in Chinese pregnant women. The purpose of this study was to assess the vitamin D status of pregnant women residing in Beijing in winter and evaluate the impact of maternal factors on serum 25-hydroxyvitamin D [25(OH)D] levels. The study was conducted on 125 healthy pregnant women. For each individual, data concerning pre-pregnancy weight, educational status, use of multivitamins and behavioral factors such as daily duration of computer use, walking and sun exposure were obtained. Serum concentrations of 25(OH)D were measured by enzyme-linked immunosorbent assay. The prevalence of vitamin D deficiency (25(OH)D < 50 nmol/L) was 96.8% and almost half (44.8%) of women were severely vitamin D deficiency (25(OH)D < 25 nmol/L). The concentration of 25(OH)D was lower in women with shorter duration of sun exposure (≤ 0.5 h/day, 25.3 ± 8.9 nmol/L) than that in women with longer duration of sun exposure (> 0.5 h/day; 30.3± 9.5 nmol/L; *P* = 0.003). Thirty six women (28.8%) had sun exposure duration ≥ 1.5h/day. The 25(OH)D concentration in these women was 31.5 ± 9.4 nmol/L which was also much lower than the normal level. Women who reported taking a multivitamin supplement had significantly higher 25(OH)D concentrations (32.3 ± 9.5 nmol/L) when compared with non-users (24.9 ± 8.2 nmol/L; *P* < 0.001). Pregnant women in Beijing are at very high risk of vitamin D deficiency in winter. Duration of Sun exposure and the use of multivitamin were the most important determinants for vitamin D status. However, neither prolonging the time of sunlight exposure nor multivitamin supplements can effectively prevent pregnant women from vitamin D deficiency. Other measures might have to be taken for pregnant women to improve their vitamin D status in winter.

## Introduction

Vitamin D is an essential fat soluble vitamin and has multiple functions. Vitamin D deficiency has been suggested to impact skeletal health as well as increase the risk of a number of non-skeletal conditions such as autoimmune diseases, cancers, type 2 diabetes mellitus, cardiovascular disease, infectious diseases, skin disorders, and schizophrenia [1]. Adequate 25(OH)D levels are particularly important for women who become pregnant, because mothers have to sustain their own vitamin D stores as well as those of their fetuses [2,3]. There is growing concern about the functional impacts of maternal vitamin D on their offspring. Low maternal levels of 25(OH)D has been suggested to be associated with some adverse outcomes for the fetus and neonate such as impaired bone development, multiple sclerosis, cancer, insulin dependent diabetes mellitus, impaired function of the immune system, asthma, and atopy [4,5]. However, evidence is inconsistent. A prospective longitudinal study showed that no associations were observed between maternal 25(OH)D concentrations and a child’s intelligence, psychological health or cardiovascular system and the concentrations of 25(OH) D in pregnancy above 75 nmol/L may result in an increased risk of atopic disorders[6]. A recent study systematically reviews randomized controlled trials and observational studies of maternal vitamin D status in pregnancy on the extraskeletal health of the offspring [7]. It is suggested that vitamin D is likely to have an effect on several offspring outcomes such as a race-dependent lowering of birthweight, type I diabetes and early childhood infections. However, data are conflicted on the impact of vitamin D on the risk of allergic conditions. There is little evidence concerning the role of maternal vitamin D in pregnancy in their child autoimmune diseases. Further investigation is needed in order to confirm the effect of vitamin D deficiency during pregnancy on various diseases in childhood. 

During the past several decades, numerous studies have reported a high prevalence of vitamin D deficiency among pregnant women in countries where women wear concealing clothing such as India [8], Saudi Arabia [9] and Iran [10] and countries in northern latitudes such as the United Kingdom [6] and Norway [11]. These women are at high risk of hypovitaminosis D, due to less sunlight exposure. Few studies have examined vitamin D status in Chinese pregnant women. Beijing (Northern China) lies at a latitude of 39.9°N. Chinese women avoid direct sun exposure to prevent from tanning their skin as fair complexion is culture preference in China. In recent years Beijing has experienced serious pollution. These factors may lead to an increase in the risk of vitamin D deficiency. Indeed, in a recent small sample size study[12], we found an extremely high prevalence of vitamin D deficiency (90%) in pregnant women in Beijing in spring. This condition could be even worse in winter, since the weather is cold and windy during winter in Beijing making outdoor activity less likely. Also, little is known about maternal factors influencing vitamin D status in Chinese pregnant women.

Generally, serum 25(OH)D levels depend on sunshine exposure, latitude, clothing habits, skin pigmentation as well as diet [13]. The risk factors for hypovitamin D could vary from place to place. There is considerable interest in factors that may influence vitamin D status in Chinese pregnant women. These include sunlight exposure and multivitamin use, and variables may relate to these factors such as income and education levels. Maternal pre-pregnant weight and pre-pregnant body mass index (BMI) are also included since adipose tissue has been shown to sequester vitamin D [14]. 

The purpose of this study was to assess the vitamin D status in pregnant women residing in Beijing in winter. In addition, we evaluated the impact of some maternal factors on serum 25(OH)D levels. The results should provide a better understanding of the maternal factors that influence vitamin D status during pregnancy and help in raising awareness as well as suggesting strategies to prevent vitamin D deficiency in China. 

## Subjects and Methods

### Subjects

Participating in this study were 125 healthy nulliparous pregnant women with singleton pregnancies. Informed consent was obtained. Pregnant women were recruited into the study at 15 - ≤ 20 weeks of gestation during their visit at the antenatal clinics of The 306 Hospital of PLA between December 2010 and February 2011. All women were local living in Beijing (latitude 39.9°N). Women with a history of any disease including renal, bone and gastrointestinal disorders and medications influencing calcium or vitamin D metabolism were excluded. 

Participants were interviewed and a data form was used to collect information about personal details, age, gestational age, pre-pregnancy weight, income, smoking and alcohol consumption. Regular use of multivitamins during the current pregnancy was also recorded. The vitamin supplements used were most commonly pregnancy-specific multivitamins, containing relatively low doses of vitamin D (between 5 and 12·5 μg/d). Vitamin D intake in food was not collected since usually little food in Chinese diet contains reasonable amounts of vitamin D. It should be noted that in China, dairy products are not fortified with vitamin D. The questionnaire also sought information about behavioral factors like daily duration of computer use and daily duration of sun exposure (duration-number of hours per day). It was assumed that only the face and hands were exposed during winter. Walking typically represented the only physical activity for these women. Physical activities therefore were recorded by the duration of walking per day (min/day) during the current pregnancy. Women were categorized by educational status as better educated women (women with associate degree or higher) and less educated women (women who did not attend university). This was based on the assumption that educational status may influence sunlight exposure, computer usage, income or multivitamin supplementation.

### Serum 25-hydroxyvitamin D measurement

Blood samples were collected from all women at 15-20 weeks of gestation. The blood samples were centrifuged and serum samples were stored at -80°Cuntil use. 

Serum concentration of 25(OH)D was determined using an enzyme-linked immunosorbent assay (ELISA) kit following the manufacturer's instructions (Immunodiagnostic Systems Ltd, Boldon, Tyne & Wear, United Kingdom). Although there has been debate over which level of serum 25(OH)D reflects optimum vitamin D status, it is generally accepted that serum 25(OH)D level of < 30ng/mL(75nmol/L) is the cut-off value for insufficiency [15-17], which is associated with maximal suppression of parathyroid hormone [18]. Vitamin D deficiency is regarded to be a 25(OH)D level < 20 ng/mL (50 nmol/L) [16,17,19] or to be < 10 ng/mL (25nmol/L) [15]. There are no data regarding optimal serum 25(OH)D levels in pregnancy so far. For the analysis, in our study we classified women into groups that defined vitamin D status: severe vitamin D deficiency: 25(OH)D < 25 nmol/L; mild vitamin D deficiency: 25 nmol/L ≤ 25(OH)D < 50 nmol/L; vitamin D insufficiency: 50 nmol/L ≤ 25(OH)D < 75 nmol/L; And sufficiency: 25(OH)D≥75 nmol/L. 

### Other measurements

All the height and weight measurements were performed by one authors. The height of each participant was measured to the nearest 0.5 cm with the subject standing barefoot and upright against a wall-mounted stadiometer. The body weight of each of the participants was measured to the nearest 0.1 kg, using an appropriate digital scale. Pre-pregnancy body mass index (BMI) was calculated using measured height and self-reported pre-pregnancy weight [weight (kg)/height (m)^2^]. 

### Ethics statement

The study design and protocol were approved by the Ethics Committee of The 306 Hospital of PLA. Ethical reference number is 2010LUNSHEN03. Written consent was obtained from all participants. 

### Statistical analysis

Statistical analysis was carried out using the SPSS statistical software package version 16.0 (SPSS Inc., Chicago, IL, USA). Data were expressed as mean ± standard deviation (SD) or number and percentage of subjects. Comparisons were conducted using unpaired Student’s two-tailed *t-test*. Chi-square tests were used to compare categorical variables. The Spearman’s Correlation Statistic was used to investigate correlations between variables. A multiple linear regression analysis was performed to determine the association between covariates and serum 25(OH)D levels. In all tests, the level of significance was P<0.05.

## Results

### General characteristics

In total, 125 healthy pregnant women in Beijing urban area participated in this study. The mean age of these subjects was 28.4 ± 2.9 years (range 20 - 37 years). Measured mean weight was 57.9 ± 7.9 kg. Pre-pregnancy BMI in most women (95.2%) was < 25. Only six women (4.8%) had pre-pregnancy BMI above 25. All women’s gravidity ranged from one to four. None of them used cigarettes or alcohol during their pregnancy. None of these women used sun screen in winter. More than three-quarters of the women had associate degrees or higher and over two thirds of women had an income above 4000 RMB (equivalent to US $650) Yuan/month/person which is roughly equivalent to the average level of monthly living cost in Beijing. About half of the women used a computer over 4 hours/day. 

### Serum 25(OH)D levels and prevalence of vitamin D deficiency or insufficiency in pregnant women

The mean serum 25(OH)D concentration of women (n 125) was 28.4 ± 9.5; (range 15.1 - 57.1) nmol/L. The percentage of vitamin D deficiency or insufficiency is shown in Table 1. The prevalence of vitamin D deficiency (25(OH)D < 50 nmol/L ) was 96.8% and no women had serum 25(OH)D concentrations ≥ 75 nmol/L which is considered as an optimal level. 

**Table 1:** Prevalence of vitamin D deficiency and insufficiency in pregnant women.

25(OH)D levels	Number	%
Severe deficiency	56	44.8
(< 25 nmol/L )		
Mild deficiency	65	52.0
(25- < 50 nmol/L)		
Insufficiency	4	3.2
(50 - < 75 nmol/L)		
Sufficiency	0	0.0
(≥75 nmol/L)		
Total	125	100.0

### Maternal characteristics and vitamin D status

Maternal characteristics, stratified by vitamin D status, are shown in Table 2. Severely deficient women were more likely to have a shorter duration of sun exposure and did not take multivitamin supplements. Better educated women were less common in the severe vitamin D deficient group than that in women with 25(OH)D ≥ 25 nmol/L. Age, gestational age, weight, height, pre-pregnant BMI, income, duration of walking and duration of computer usage were not different between the two groups. 

**Table 2:** Maternal characteristics of all subjects by vitamin D group.

Descriptive	Women with 25(OH)D<25 nmol/l	Women with 25(OH)D≥25 nmol/l	p
Number (n, %)	56 (44.8)	69 (55.2)	___
Age (years, SD)	28.1 (2.67)	28.7 (3.02)	P = 0.206
Gestational age (weeks, SD)	17.0 (1.26)	16.8 (1.11)	P = 0.239
Height (cm, SD)	161.9 (3.26)	161.2 (4.03)	P = 0.326
Weight (kg, SD)	58.3 (8.37)	57.6 (7.62)	P = 0.616
Pre-pregnant weight (kg, SD)	53.4 (6.81)	52.9 (6.65)	P = 0.648
Pre-pregnant BMI (kg/m^2^, SD)	20.4 (2.49)	20.3 (2.34)	P = 0.902
Assoc. degree or higher (n, %)	38 (67.9)	59 (85.5)	P = 0.019
Income (RMB thousand Yuan/month/person, SD)	5.10 (2.69)	4.62 (2.28)	P = 0.289
Multivitamin users (n, %)	13 (23.2)	45 (65.2)	P < 0.001
sun exposure (h/day, SD)	0.76 (0.71)	1.08 (0.68)	P = 0.014
Computer usage ((h/day, SD)	3.96 (2.56)	3.64 (2.46)	P = 0.494
Walking (min/day, SD)	137.5 (94.47)	133.3 (83.18)	P = 0.796

### The influence of maternal factors on vitamin D status

**Figure 1:**
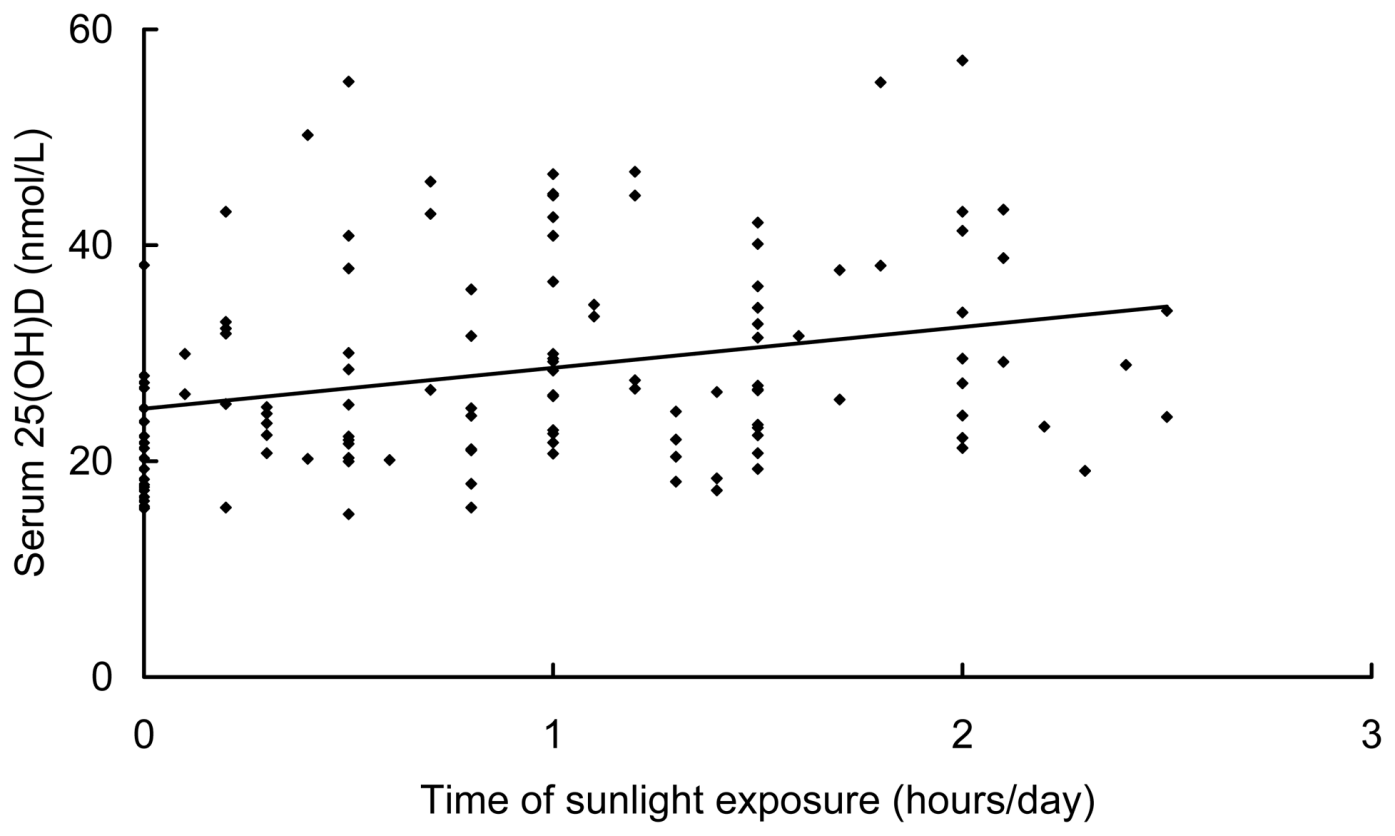
The correlation of serum 25(OH)D and time of sun exposure. Scatter plot showing the relations between serum 25(OH)D concentrations and time of sun exposure (n = 125; r = 0.287, p = 0.001).

Forty eight women (38.4%) had sun exposure time ≤ 0.5 h/day. The percentage of severe vitamin D deficiency was much higher in women with shorter duration of sun exposure (≤ 0.5 h/day; 58.3%) than that of women with longer duration of sun exposure (> 0.5 h/day; 36.4%). Serum 25(OH)D concentration showed a significantly positive correlation with the time of sun exposure (r = 0.332, *P* < 0·001; Figure 1). The concentration of 25(OH)D was lower in pregnant women with shorter duration of sun exposure (≤ 0.5 h/day, 25.3 ± 8.9 nmol/L) than that in women with longer duration of sun exposure (> 0.5 h/day; 30.3 ± 9.5 nmol/L; *P* = 0.003). Thirty six women (28.8%) had a sun exposure duration ≥ 1.5 h/day. The 25(OH)D concentration in these women (31.5 ± 9.4 nmol/L) were still very low. None of these women used sunscreen during the time period of this study.

Of the 125 pregnant women recruited, 58 (46.4%) were multivitamin supplement users. The amount of vitamin D contained in multivitamin supplements consumed ranged from 5 to 12.5 μg. Amongst supplement users, 13/58 (22.4%) were severely vitamin D deficient *vs.* 43/67(64.2%) of non-users (*P* < 0·001). Student’s *t*-test analysis showed that pregnant women who reported taking a multivitamin supplement had significantly higher 25(OH)D concentrations (n = 58, 32.3 ± 9.5 nmol/L) when compared with women who did not take multivitamin supplements (n = 67, 24.9 ± 8.2 nmol/L; *P* < 0·001). 

To determine whether education levels impact vitamin D status, women were grouped by better educated women (with associate degree or higher) and less educated women. Serum 25(OH)D concentration was higher in better educated women (n = 97, 29.7 ± 10.0 nmol/L) than in less educated women (n = 28, 24.0 ± 5.9 nmol/L; *P* < 0·001). The percentage of severe vitamin D deficiency was much lower in better educated women (38/97, 39.2 %) than that in less educated women (18/28, 64.3%). 

In multiple regression analysis, multivitamin supplement use (coefficient β = 0.357; *P* < 0.001) and sun exposure (coefficient β = 0.200; *P* = 0.018), but not education status, were significantly associated with 25(OH)D levels.

Age, weight, pre-pregnant weight, pre-pregnant BMI, gestational age, income, duration of walking, and duration of computer use were not correlated with 25(OH)D concentrations. 

### The influence of education levels on sun exposure and multivitamin use

The duration of sun exposure was longer in better educated women (1.08 ± 0.65 hours/day) than that in less educated women (0.44 ± 0.68 hours/day; *P* < 0·001). Also, there were more multivitamin users in better educated women (53/97, 54.6%) when compared with less educated women (5/28, 17.9%; *P* = 0.001). 

## Discussion

The present study showed a high prevalence of vitamin D deficiency among pregnant women in the Beijing urban area (39.9 °N), northern China in winter. Over 90% of women had vitamin D deficiency (< 50nmol/l) and none of them reached normal 25(OH)D concentrations (≥ 75 nmol/L). The study confirmed the results from our previous study which was conducted in spring [12]. These studies suggest that pregnant women in Beijing are at very high risk of vitamin D deficiency in either winter or spring.

Other studies have demonstrated that vitamin D deficiency is common in pregnant women in different countries, such as 31% in southern India [8], 19.5% in Greece [20] and 5.7 % in Iran [10]. One study observed the vitamin D status in pregnant women of several ethnic backgrounds who reside in The Hague in the Netherlands (52° N). Of all subjects, 8% of Western people and most non-Western subjects (59%-84%) had vitamin D deficiency [21]. A study from Chengdu urban area (30.7°N; Southwest China) showed that the prevalence of vitamin D deficiency was 57.1% in pregnant women in spring [22]. Direct comparisons between studies are not possible due to the use of different methods and inconsistent definitions of vitamin D deficiency. It seems, however, that all pregnant women are at a high risk of vitamin D deficiency. The predominant risk factors for hypovitamin D could vary from place to place, though, generally, serum 25(OH)D levels depend on sunshine exposure, latitude, clothing habits, skin pigmentation, and diet [13]. 

The major source of vitamin D for humans is exposure of the skin to sunlight [17]. Thus, keeping enough sunlight exposure is important to prevent vitamin D deficiency. In our study the results showed that the concentration of 25(OH) D is positively associated with the duration of sun exposure and this is consistent with previous reports [8]. Women with duration of sun exposure less than 0.5 h/day had higher rates of severe vitamin D deficiency. This suggests that sun exposure plays a role in maintaining vitamin D levels in Chinese pregnant women in winter. In the study, 38.4% of women had sun exposure less than 0.5 h/day. In Beijing the weather is cold and windy in winter. These conditions tend to reduce the time that women spend outdoors and lead to a decrease in UV-B exposure. In addition, for cultural reasons Chinese women avoid direct sun exposure to keep their skin fair. Therefore it is necessary to educate people about the knowledge of vitamin D, especially for pregnant women. Longer duration of sun exposure may be required for Chinese pregnant women.

How much sun exposure results in adequate vitamin D synthesis? It has been suggested that in sun-rich Brisbane, Australia, between 2 and 4 minutes sun exposure per day was needed during summer months, at noon, for a Caucasian to produce enough vitamin D[23]. The exposure duration required is highly dependent on skin pigmentation. Previous experiments suggest that Indians require about three times the UV exposure as white Caucasians to produce the same amount of vitamin D [24]. Season and latitude could also influence the duration of sunlight exposure required [25]. In Beijing (39.9°N), China, how much sunlight exposure is needed for women to form sufficient vitamin D in winter? The present study showed that even with sun exposure time over 1.5h/day, the 25(OH) D concentration was still very low. This may suggest that the role of sun exposure is limited in elevating vitamin D concentration in pregnant women residing in Beijing in winter. In addition, the cold weather in Beijing also limits the area of skin exposed. Normally, in winter, only the face and hands can be exposed in Beijing. It has been reported that minimal skin exposure of arms and face will only result in a nominal production of vitamin D-3 [26]. This may explain why prolonging sun exposure time did not effectively increase 25(OH)D level in our study. 

Solar zenith angle (the angle that sunlight enters the atmosphere) affects the amount and distribution of solar radiation reaching the earth’s surface [27]. Increasing solar zenith angle results in more of the UVB radiation being absorbed by the ozone layer and little reaching the Earth's surface. In addition, increasing solar zenith angle could also enlarge the area which sunlight spread and thus decrease its intensity. The angle can be changed due to changing latitude, season, and time of day [28]. Above 37° latitude, during the fall and winter months, the zenith angle of the sun is oblique, and therefore the solar UV-B photons are efficiently absorbed by the ozone layer resulting in little cholecalciferol being made in the skin [29]. Vitamin D production in human skin occurs only when incident UV radiation exceeds a certain threshold. Matsuoka et al. demonstrated that a UV-B irradiation threshold of 18 mJ/cm^2^ was required to induce vitamin D-3 production for healthy white subjects [30]. However, the exposure level of 18–20 mJ/cm^2^ is not generally reached during the winter above latitude 40° and a Caucasian individual in a bathing suit outside on a sunny January day would not produce endogenous vitamin D-3 [29]. Therefore, people living in high latitudes would not achieve sufficient vitamin D from sunlight exposure in winter. It has been reported that women in high latitudes have more vitamin D insufficiency [31]. Beijing lies at a latitude of 39.9 degrees north. It is not surprising that there is a high prevalence of vitamin D deficiency in pregnant women residing in Beijing in winter.

In addition, pollution may be also a factor that affects UV-B reaching the skin and reducing endogenous vitamin D production in the skin. Beijing has experienced serious pollution in recent years. It has been suggested that air pollution is one of the main factors in determining the percentage of the ground level of UVB [32]. One study in India has found that high atmospheric pollution decreases the percentage of UVB which reaches the earth surface and children in more polluted areas are at higher risk of vitamin D deficiency [33]. Other studies have also shown that the air pollution has a significant influence on vitamin D status [34,35]. Women in Beijing can not produce enough vitamin D through sunshine exposure in winter due to multiple factors. Therefore other effective measures may need to be taken to improve vitamin D status in Chinese pregnant women. 

It has been reported that vitamin D supplementation can effectively improve vitamin D status [36]. A recent randomized clinical trial in non-western immigrants in the Netherlands (none Asian) found vitamin D supplementation to be more effective than advised sunlight exposure for treating vitamin D deficiency [37]. In our study nearly half of women did take multivitamins and these multivitamins did improve the vitamin D level somewhat but did not prevent vitamin D deficiency. The supplements in our study used were most commonly pregnancy-specific multivitamins, containing relatively low doses of vitamin D (between 5 and 12·5 μg/d), obviously, which does not effectively alter the outcome of high prevalence of vitamin D deficiency. Appropriate dose of vitamin D supplementation for women may be required. 

Vitamin D supplementation in pregnant women has been recommended in some countries. In Canada, the adequate intake for vitamin D in pregnancy is 5 μg/d [38], while in the UK the reference nutrient intake is set at 10 μg/day [39]. Apparently, these recommended dosages do not seem adequate for Chinese pregnant women in winter. Higher amounts of vitamin D may be required, though the precise dose remains unknown. One study [40] recruited different races of women at 27 weeks gestation. Either Single (200,000 IU vitamin D) or daily (800 IU) dose of vitamin D has been effective at raising circulating 25(OH)D concentrations. However, even with supplementation, only a small percentage of women and babies were vitamin D sufficient. Research in adults suggests that a daily dietary allowance of 1000-2000 IU/day is needed to achieve a target circulating 25(OH)D value of at least 75 nmol/L [41]. However, in pregnant women, a study evaluating plasma vitamin D status has shown that vitamin D supplementation of <2000 IU/day is not effective in achieving sufficiency [42]. Another study showed that vitamin D supplementation of 4000 IU/day for pregnant women is most effective in achieving sufficiency in all women regardless of race [43]. In 2011, the US Endocrine Society endorsed the need for at least 600 IU(15 ug)/ day of vitamin D supplementation for women during pregnancy and up to 1500–2000 IU/day to maintain 25(OH)D blood levels above 30 ng/mL [16]. There is no regular vitamin D supplementation in China at this time. A hundred percent of pregnant women had vitamin D insufficiency in our study. On the basis of these findings, it seems logical that pregnant women should routinely be supplemented with vitamin D in China. However, since there is insufficient data to evaluate the effectiveness of vitamin D supplementation during pregnancy vitamin D supplementation as routine antenatal care is yet to be determined. Further high quality randomized trials are required to evaluate the safety and effectiveness of vitamin D supplementation in pregnancy.

In the present study, no women intended to use vitamin D and only 46.4% of women reported taking pregnancy-specific multivitamin supplements. A survey revealed considerable ignorance and confusion about the function of vitamin D and sources of vitamin D in Chinese women [44]. Measures should be taken to improve their knowledge about vitamin D. Treatment should be paired with health education and advice and basic knowledge of the role of vitamin D. 

In the study, the 25(OH) D level was low in all subjects, but was particularly low in less educated women. Better educated women had higher serum 25(OH)D concentrations and lower rates of severe vitamin D deficiency. In addition, the group of better educated women had more multivitamin users and longer duration of sun exposure than the group of less educated women. It has also been reported that the women using dietary supplements during pregnancy is associated with education [45]. Evidence shows that people with more education are likely to have greater knowledge of health conditions and are more likely to engage in healthy behaviors [46]. This suggests that education levels are most likely to influence women’s health awareness rather than directly affect vitamin D status. 

Obesity is a risk factor for vitamin D deficiency [47]. It has been reported that pre-gravid obese women had significantly lower serum 25(OH)D concentrations and dose-dependent relations between pre-pregnancy BMI and maternal vitamin D status [48]. In the present study, 25(OH)D concentration showed no association with pre-pregnancy BMI. This could be because the majority of women in our study had normal pre-pregnancy BMI. Only 4.8% of women had pre-pregnancy BMI over 25 and no women were obese (BMI ≥30.0). Therefore, there were no enough subjects who were over weight or obese for analysis of the impact of this factor on vitamin D status. In addition, self-reporting pre-pregnancy weight may influence the accuracy of the result analysis.

The limitation of this study is that all subjects were from the Beijing urban area. Their educational status and/or behavior may be different from rural areas. Therefore, the results could not represent the condition of rural women in China. Also, the study lacked dietary information. Therefore we may not analyze the possibility that dietary factor contributed to our results.

To our knowledge, this study is the first to report the influence of maternal factors on vitamin D status in Chinese pregnant women. This design allowed us to assess the impact of these factors on vitamin D status in Chinese pregnant women. The effect of prolonging the duration of sun exposure on maintaining appropriate vitamin D levels is limited in pregnant women residing in Beijing in winter. Importantly, we did have the information on multivitamin supplement usage, which allowed us to show that while pregnancy-specific multivitamin supplements improve the status in women at low risk of severe vitamin D deficiency, it does not prevent women from vitamin D deficiency in winter. Measures may have to be taken to improve vitamin D status in pregnant women in China.

## Supporting Information

File S1 **Collected data.**
(XLS)
